# Health state descriptions to elicit stroke values: do they reflect patient experience of stroke?

**DOI:** 10.1186/s12913-014-0573-6

**Published:** 2014-11-21

**Authors:** Joanne Gray, Mabel L S Lie, Madeleine J Murtagh, Gary A Ford, Peter McMeekin, Richard G Thomson

**Affiliations:** School of Health, Community and Education Studies, Northumbria University, Coach Lane, Benton, Newcastle upon Tyne, NE7 7XA UK; Institute of Health and Society, Newcastle University, Newcastle Upon Tyne, UK; Institute of Cellular Medicine, Newcastle University, Newcastle Upon Tyne, UK; Institute for Ageing and Health (Stroke Research Group), Newcastle University, Newcastle Upon Tyne, UK; School of Social & Community Medicine, Bristol University, Bristol, UK

**Keywords:** Cerebrovascular disease/stroke, Outcome research, Quality of life, Preference elicitation, Patient experience

## Abstract

**Background:**

To explore whether stroke health state descriptions used in preference elicitation studies reflect patients’ experiences by comparing published descriptions with qualitative studies exploring patients’ lived experience.

**Methods:**

Two literature reviews were conducted: on stroke health state descriptions used in direct preference elicitation studies and the qualitative literature on patients’ stroke experience. Content and comparative thematic analysis was used to identify characteristics of stroke experience in both types of study which were further mapped onto health related quality of life (HRQOL) domains relevant to stroke. Two authors reviewed the coded text, categories and domains.

**Results:**

We included 35 studies: seven direct preference elicitation studies and 28 qualitative studies on patients’ experience. Fifteen coded categories were identified in the published health state descriptions and 29 in the qualitative studies. When mapped onto domains related to HRQOL, qualitative studies included a wider range of categories in every domain that were relevant to the patients’ experience than health state descriptions.

**Conclusions:**

Variation exists in the content of health state descriptions for all levels of stroke severity, most critically with a major disjuncture between the content of descriptions and how stroke is experienced by patients. There is no systematic method for constructing the content/scope of health state descriptions for stroke, and the patient perspective is not incorporated, producing descriptions with major deficits in reflecting the lived experience of stroke, and raising serious questions about the values derived from such descriptions and conclusions based on these values.

## Background

Health related quality-of-life assessment methods are increasingly used to develop indices that can support health economic evaluation of stroke care. Utility (or preference-based) measures, typically reporting on a single 0 to 1 scale, based upon decision and utility theories, are designed to elicit the value people place on a particular health state. Such preference-based approaches integrate different aspects of health into a single index, usually anchored by a value of ‘1.00’ for full health and ‘0’ for death. These measures are increasingly important since they are used to actively inform both health policy and individual decision-making. At a population level, they support resource allocation decisions with their use in health technology assessment and economic evaluation that lead to the production of guidance to health services regarding the use of health interventions e.g. in England and Wales guidelines are produced by National Institute for Health and Care Excellence (NICE) [1]. Furthermore, these measures are adopted in guideline production in a number of different countries including Australia [2] Canada [3] and the United States [4]. They have also been used to support decision making at an individual level, e.g. by incorporating decision analytical models in clinical decision support systems and patient decision aids [5,6].

Two approaches to utility elicitation exist - indirect and direct. In indirect elicitation patients complete a questionnaire, such as the EQ-5D, which is mapped onto utility scores previously developed. Indirect methods are less time consuming for respondents, but lack content coverage and are often insensitive to change [7-9]. Direct methods are more complex and time consuming, but it is suggested that they may be more reliable, valid and responsive [10]. They require health state descriptions for specific health states such as major or minor stroke (examples are shown in Table 1), and derive a value by taking respondents through a valuation exercise, such as visual analogue scale (VAS) standard gamble (SG) or time trade off (TTO) [11,12]. These approaches differ in the way they are undertaken, but all seek to derive a value between 0 and 1 for the relevant health state by taking people through a guided exercise.

**Table 1:** **Examples of health state descriptions**

**Author**	**Health state description**
Robinson et al. 2001 [13]	**Mild stroke**
	• Your arm and leg are a little weak on one side
	• Your speech is a little slurred but people understand you
	• You may be unable to perform some of your usual activities
	• You can look after yourself as usual
	**For the rest of your life**
	**Severe stroke**
	• One side of your body is totally limp (paralysed)
	• Your speech is slurred – it is very hard to understand you
	• You are unable to perform most of your usual activities
	• You cannot look after yourself without help
	**For the rest of your life**
Hallan et al. [14]	**Minor stroke (Rankin scale: level 2–3)**
	• Your right arms is limp (paralysed) and your leg is slightly weakened
	• You can think, read and speak clearly
	• You have full control of bladder and bowel
	• You can walk at normal speed, but with a slight limp
	• You must learn to write with the left arm
	• You need some help with feeding, dressing and other tasks normally requiring both arms
	**Major stroke (Rankin scale: level 4–5)**
	• The right side of your body is totally limp (paralysed)
	• You can think clearly
	• Your speech is slow and unclear but understandable
	• You have full control of bladder and bowel
	• You cannot walk at all so you must use a wheelchair
	• You need some help for feeding, dressing and transferring
	**You are totally dependent on help for bathing**
	**You may need to go to a nursing home**

Equally, it is increasingly recognised that health services and policy need to reflect the patient and public perspective through patient centred and personalised care [15]. This implies that any such values derived from patients and the public, and used to support decision-making, should be valid and reliable, and appropriately reflect lived patient experience.

It is widely reported that direct utility estimates vary considerably, which might reflect the method used or the way the health states are described [16]. Specifically for stroke, variations in estimates resulting from direct preference elicitation have previously been explained by a number of factors. First, the choice of study population; for example, healthy participants assign lower utilities than patients who have experienced stroke [11,17]. Second, the method of elicitation, with standard gamble derived scores being generally higher than those derived from time trade off studies, which in turn are higher than those derived from visual analogue scales [16,18]. Third, the bounds of the scale, typically 0–1, may be defined differently: the upper bound defined as perfect health yields lower utility scores than if defined as the absence of the condition of interest, which is no guarantee that health is perfect [19].

Variations in health state descriptions content may also explain differences. The level of detail in health state descriptions can affect utility scores; longer, more comprehensive, descriptions (although appearing to have more face validity), can overload respondents’ cognitive capacity so that they latch onto a few key phrases and ignore the rest [20]. Naming or labelling a condition may have an impact [20-23] with a recent study recommending avoiding condition labels in health state descriptions to ensure that values are not affected by prior knowledge or preconceptions of the condition that may distort the health state being valued [10]. Furthermore, the wording may also cause variation in values if not presented in a balanced manner (framing bias), with both positive and negative effects described; explicit inclusion of negative aspects of stroke elicit lower values [24,25].

Despite this, little attention has been given to the appropriateness of health state descriptions. If they don’t adequately reflect the reality of the health states they seek to measure, decisions based on these derived estimates may be compromised. Hence, at the very least, descriptions should reflect the reality of living with a stroke. Therefore, we set out to determine whether published stroke health state descriptions used in value elicitation studies truly reflect patients’ experiences of stroke by comparing their content with the results of qualitative studies exploring patients’ lived experience of stroke.

## Methods

Two literature reviews were conducted: stroke health state descriptions used in direct preference elicitation studies and the qualitative literature on patients’ experience of stroke. The former review sought to comprehensively capture published health state descriptions for stroke used in preference elicitation studies; the latter, to capture what is important to patients in their lived experience of stroke from qualitative studies of patient perspectives. Both reviews involved the use of search strategies that included a combination of both subject headings and relevant key words.

### Search strategy for health state descriptions

MEDLINE, CINAHL and PsychInfo were searched (1980 to 2011) using the search terms: stroke, cerebrovascular accident, cerebral arterial diseases, cerebrovascular disorders, cerebral thrombosis, carotid artery thrombosis, cerebral haemorrhage, cerebral hematoma, apoplexy, hemiplegia and hemiparesis. These terms were combined with the following terms: utility, quality of life, preference elicitation, time trade off (TTO), standard gamble (SG), Quality Adjusted Life years (QALY), stroke preferences, cost-effectiveness analysis and cost-utility analysis. Reference lists of all included articles were also searched.

Articles were included if they used appropriate direct preference elicitation methods, were in English and included the wording of the health state description. One reviewer performed initial selection (JG). Two reviewers (ML and RT) independently assessed studies for inclusion and extracted data, with disagreement resolved by discussion.

### Search strategy for patients’ experience of stroke

MEDLINE, Embase, ISI Web of Knowledge and PsychINFO were searched (1997 to 2011) using the search terms: stroke, experience and qualitative, supplemented by hand searching reference lists from papers identified in both searches and related PubMed citations. Studies were included if: the findings focused on experiences of patients with stroke; were in English; used accepted qualitative methods; provided a clear exposition of methods and data collection; were supported by direct quotations; and were deemed of sufficient quality. Quality of the studies was based on criteria established by Mays and Pope [26]. Articles were scrutinised by two experienced qualitative researchers (MM, ML) and were included in the review if they were based on the appropriate application of established qualitative methods –that data collection, analysis and sample selection were appropriate to the explicit or implicit research question(s) - and contributed to knowledge in the field. Studies that did not provide sufficient detail of methods or those reporting opinion, but not providing direct empirical evidence, were rejected. Articles on the experiences of carers and professionals, trial participation, and assessments of rehabilitation therapies, information provision, and healthcare and community services were excluded. Articles focusing on specific characteristics of stroke experience such as end of life, pain, communication, return to employment or wheelchair use were excluded to avoid the data being skewed to one particular aspect of the stroke experience. Results and discussion sections were extracted for use in content and thematic analysis. The extracted data were coded as described below.

### Content analysis and interpretation

Content analysis [27-29] was used by MLSL to identify the characteristics of stroke experience included in health state descriptions and in the results/discussion sections of qualitative studies. Using an inductive approach, text describing the patient experience was subjected to open-coding and category creation with the help of NVIVO software [30]. A comparative analysis was conducted to ensure the distinctions between the categories and the consistency of the content coded within each of the categories as well as across the two sets of data sources i.e. preference elicitation studies and qualitative literature. Qualitative inter-rater checking of definitions of the categories and the coded text was carried out by JG and discussed by the research team. Data regarding counts of the number of studies that included each category was extracted in order to undertake a comparative analysis of both types of study in terms of these categorised counts. The categories and counts of study were further mapped onto four predefined domains of HRQOL that encompass relevant areas from the stroke patients’ perspective as being crucial to quality of life measurement [7,30]: biophysical, mood and cognition, prognosis and social domains.

In order to interpret the experience of stroke represented by the characteristics defined in the content analysis we also undertook a thematic synthesis of the qualitative studies [31]. This method includes systematic searching of the literature, quality assessment (as described above), extraction of data and thematic analysis of those data, i.e. familiarisation and coding line by line to develop descriptive themes. The text of results sections of each qualitative article formed the data for analysis. The thematic analysis conducted by MJM was an inductive process which followed the six stages described by Baun and Clarke [32]: familiarisation, generation of initial codes, searching for themes, reviewing themes, defining and naming themes and reporting the analysis. Here we term these ‘categories’ for ease of comparison with the content analysis. Content and thematic analyses of categories are integrated in the results below. Interpretation of these themes forms the analytic component of the analysis and is reported in the discussion.

## Results

### Health state descriptions

Seven studies that used direct preference elicitations were included (Table 2) [13,14,17,24,33-35]. Research participants included stroke survivors, those at increased risk of stroke and healthy people. Health descriptions ranged from mild/minor, moderate to severe/major stroke, examples of which are presented in Table 1, with standard gamble and time trade off the most frequent methods.

**Table 2:** **Summary of preference elicitation studies**

**Preference elicitation study**	**Objective**	**Research subjects**	**Types of stroke covered by health state descriptors**	**Development of health state descriptions: information sources**	**Method of elicitation**
Solomon et al. 1994 [24]	To examine patient preferences for different outcomes of stroke including death	All outpatients referred to a neuro-diagnostics laboratory for ultrasound evaluation of the carotid artery	Consequences of stroke: mild, moderate and severe impairment of three types of neurological deficit: motor, language and cognitive. Descriptions for a painless fatal stroke and perfect health.	Stroke deficit types scaled in terms of severity classifications: mild, moderate and severe impairment. Scaling validity of stroke deficit types: tested by three neurologists specialized in stroke care.	Rank and scale method over a 100 point range: 100 representing perfect health and 0 representing the worst possible health state.
		Age, y(mean ± SD ): 73 ± 9			
		Gender, % female: 45			
		Country: USA			
				No reference to how or why deficit types were identified	
Gage et al. 1996 [33]	To determine how stroke and stroke prophylaxis affect quality of life using direct preference elicitation	Patients with atrial fibrillation, at least 50 years of age, could read English and who did not reside in a convalescent hospital	Mild, moderate and major stroke	Categorised by progressively more severe neurological deficit based on Modified Rankin Scale (mild - mRS 1 or 2, moderate 3 or 4, severe 4 or 5). Utilised van Hoeyweghen et al. [36] which recommended that stroke descriptions of function cover multiple domains: fine and gross motor skills, spoken and written language are, and cognitive and psychosocial function	Time trade-off and standard gamble
		Age, y(mean ± SD ): 70.1 ± 7.3			
		Gender, % male: 86			
		Country: USA			
Shin et al. 1997 [34]	To determine younger patients’ perceptions of quality of life with a stroke by eliciting utility values	Younger patients with arteriovenous malformations who are at risk of a stroke or have experienced one.	Major and minor stroke	No information regarding how stroke severity classifications were developed	Standard gamble
		Age, y(mean)(range): 37(18–57)			
		Gender: not reported			
		Country: Canada			
Samsa et al. 1998 [17]	To examine attitudes toward hypothetical major stroke	Patients at increased risk of stroke including those with and without a history of cerebrovascular symptoms but at increased risk of stroke due to conditions such as atrial fibrillation, hypertension and vascular heart disease	Major stroke with and without aphasia	No information regarding how stroke severity classifications were developed	Time trade-off
		Age, y(mean): 65			
		Gender, % male: 52			
		Country: USA			
Hallan et al. 1999 [14]	To elicit valid quality of life estimates and the highest acceptable treatment risk of different outcomes after stroke	Healthy people, non stroke medical patients and stroke survivors 20–84 years old	Minor and major stroke	Classifications for minor and major stroke based on Rankin scale 2–3 and 4–5 respectively	Standard gamble, time trade-off and direct scaling
		Age, y(mean): not reported			
		Gender: not reported			
		Country: Norway			
Robinson et al. 2001 [13]	To elicit patient valuations of health states relevant to the assessment of the prevention of stroke by warfarin anticoagulation therapy	Patients over the age of 60 years with atrial fibrillation	Mild and severe stroke as well as hospital managed warfarin and major bleed	Adapted from 2 previous studies	Standard gamble
		Age, y(mean)(range): 73(60–87)			
		Gender, % male: 54			
		Country: England			
Slot and Berge 2009 [35]	To ascertain patients’ preferences for thrombolytic treatment for acute stroke	Elderly people at five day care centres: ischaemic stroke survivors and age- matched control subjects who were at risk of stroke	Mild, moderately severe and severe ischaemic stroke	Based on Modified Rankin Scale for mild (mRS =1), moderately severe (mRS =3) and severe (mRS = 5) stroke	Standard gamble
		Age, y(mean ± SD): 78 ± 6			
		Gender: not reported			
		Country: Norway			

### Patients’ experience of stroke

Twenty eight qualitative studies examining post stroke experience were included [37-64] (Table 3) and were conducted in the UK (n = 9), the USA (n = 7), Norway (n = 4), Sweden (n = 4), Canada (n = 3) and Australia (n = 1). Methods predominantly comprised one-to-one interviews, with two studies employing focus groups [37,38]. The theoretical perspectives for analysis included phenomenology [39-46], grounded theory [37,39,47-49], narrative analysis [50-52] and discourse analysis [53]. Participants were predominantly over 60 and Caucasian, although US studies included Hispanic and African Americans. Two studies [49,51] included a small number of Bangladeshis and African Caribbeans. Specific sub-populations were targeted in seven studies: elderly non-institutionalised women [54], women in a rural setting [41], patients in the community [55], working class men [43], young women stroke survivors [56] and US war veterans [47,52].

**Table 3:** **Summary of qualitative studies**

	**Author & date**	**Title of article**	**Country**	**Sample characteristics**	**Details**
1	Nilsson I, Jansson L, Norberg A. 1997 [45]	To meet with stroke: Patients’ experiences and aspects seen through a screen of crises.	Sweden	n =10	Narrative interviews one month and two months after discharge. Phenomenological hermeneutic analysis.
				9 male, 1 female	
				Age: 53-81	
2	Pound P, Gompertz P, Ebrahim S. 1998 [49]	Illness in the context of older age: The case of stroke.	UK	n =40	In-depth semi-structured interviews. Grounded theory and constant comparison.
				21 male, 19 female	
				Age: 40-87	
				Predominantly working-class elderly	
3	Pound P, Gompertz P, Ebrahim S. 1998 [57]	A patient-centred study of the consequences of stroke.	UK	As above	As above
4	Wyller, T.B; Kirkevold, M. 1999 [58]	How does a cerebral stroke affect quality of life? Towards an adequate theoretical account.	Norway	n =6	Interviewed three years after stroke. Thematic analysis
				4 male, 2 female.	
				Age: 65-85	
5	Pilkington F. 1999 [59]	A qualitative study of life after stroke.	Canada	n =13	32 interviews at 3 time points: during acute stay, 1 month and 3 months after stroke. Longitudinal descriptive exploratory analysis.
				9 male, 4 female	
				Age: 40-91	
6	Secrest J, Thomas S. 1999 [46]	Continuity and discontinuity: the quality of life following stroke.	US	n =14	Interviewed between nine months and 23 years after stroke. Existential phenomenological methodology.
				7 male, 7 female	
				Age: 40-93	
7	Ellis-Hill CS, Payne S, Ward C. 2000 [51]	Self-body split: Issues of identity in physical recovery following a stroke.	UK	n =8	Life narrative approach, interviews during hospital stay, 6 months and one year post-discharge. Twenty four interviews in total.
				5 male, 3 female	
				Age: 56-82	
8	Bendz M. 2000 [53]	Rules of relevance after a stroke	Sweden	n =10	Interviews three to four months after incident. Medical records also analysed. Discourse analysis.
				6 male, 4 female	
				Age: 58-65	
				1^st^ time stroke survivors	
9	Dowswell GP, Lawler JP, Dowswell TP, Young JF, Forster AP, Hearn JP. 2000 [60]	Investigating recovery from stroke: A qualitative study.	UK	n =30	Interviews after an RCT, 13–16 months post-stroke. Thematic analysis.
				stroke patients	
				15 caregivers	
10	Burton CR. 2000 [39]	Living with stroke: A phenomenological study.	UK	n =6	Tracked for 12 months after stroke. 73 interviews in total. Phenomenology and grounded theory methods.
				2 male, 4 female	
				Age: 52-81	
11	Eaves YD. 2000 [50]	‘What happened to me’: Rural African American elders’ experiences of stroke	US	n =8	Descriptive narrative analysis.
				2 male, 6 female	
				Age: 56-79	
				African American elders	
				10 care-givers	
12	O’Connell B, Hanna B, Penney W, Pearce J, Owen M, Warelow P. 2001 [38]	Recovery after stroke: A qualitative perspective.	Australia	Stroke survivors	Five focus groups, three with stroke survivors, 2–180 months after stroke, one with carers, and one with key informants. Total of 40 participants. Content analysis
				Age: 20-89	
				Carers and key informants	
13	Kirkevold M. 2002 [61]	The unfolding illness trajectory of stroke.	Norway	n =9	63 interviews. First interview 1–2 weeks after onset. Prospective and longitudinal case studies
				mild to moderately affected stroke patients	
14	Hilton E. 2002 [54]	The meaning of stroke in elderly women: a phenomenological investigation.	US	n =5	Interviewed twice in non-institutionalised settings at least 1 year post-stroke. Hermeneutic phenomenology.
				Elderly women	
				Age: 66–80 years	
15	Gubrium JF, Rittman MR, Williams C, Young ME, Boylstein CA. 2003 [62]	Benchmarking as everyday functional assessment in stroke recovery.	US	Male stroke survivors of various ages and from three ethnic groups (Hispanic, African American, and non-Hispanic White)	40 in-depth qualitative interviews one month following discharge
16	Kvigne K, Kirkevold M. 2003 [41]	Living with bodily strangeness: Women’s 17experiences of their changing and unpredictable body following a stroke.	Norway	n =25	Interviewed three times: during 1^st^ 6 weeks, 6 months and one year post-stroke. Phenomenological and feminist study.
				25 female	
				Age: 37-78	
				Women in rural Norway	
				17 partnered	
17	Kvigne K, Kirkevold M, Gjengedal E.2004 [42]	Fighting back - struggling to continue life and preserve the self, following a stroke.	Norway	As above	As above
18	Murray CD, Harrison B. 2004 [44]	The meaning and experience of being a stroke survivor: an interpretative phenomenological analysis.	UK	n =10	5 interviewed, 5 corresponded by e-mail. Averaged 9 years post-stroke. Interpretative Phenomenological Analysis (IPA)
				4 male, 6 female	
				Mean age: 48.8 years	
19	Carlsson G, Möller A, Blomstrand C. 2004 [48]	A qualitative study of the consequences of ‘hidden dysfunctions’ one year after a mild stroke in persons <75 years.	Sweden	n =15	Interviews analysed with grounded theory
				8 male, 7 female	
				Age: 30-69	
				Patients with mild stroke living with spouse	
20	Faircloth CA, Boylstein C, Rittman M, Gubrium JF. 2005 [52]	Constructing the stroke: Sudden-onset narratives of stroke survivors.	US	n =111	In-depth interviews. Data collected at months1, 6, 12, 18 and 24 after discharge, but only data from 1, 6, and 12 reported here. Narrative interpretive method.
				Male veterans	
				Average age: 67	
				From 3 ethnic groups: Puerto Rican Hispanic; African American, and non-Hispanic White.	
21	Clarke P, Black SE. 2005 [55]	Quality of life following stroke: Negotiating disability, identity, and resources.	Canada	n =8	Interviewed 7 months to 8 years post stroke. Selected principles of grounded theory used.
				3 male, 5 female	
				Age: 60 and above	
				Living in a community dwelling	
22	Lobeck M, Thompson AR, Shankland MC. 2005 [43]	The experience of stroke for men in retirement transition.	UK	n =7	Interviewed more than 6 months post-stroke. Interpretative Phenomenological Analysis.
				7 male	
				Age: 64-70	
				From a working class background.	
23	Stone SD. 2005 [56]	Reactions to invisible disability: The experiences of young women survivors of hemorrhagic stroke.	Canada	n =22	Open ended in-depth interviews. Constant comparison method.
				22 female	
				Age: 8–49 at the time of stroke	
				Age: 19–57 at the time of interview	
				From four different countries: Scotland, England, U.S. and Canada, majority Caucasian	
24	Olofsson A, Andersson SO, Carlberg B. 2005 [63]	‘If only I manage to get home I’ll get better’-Interviews with stroke patients after emergency stay in hospital on their experiences and needs.	Sweden	n =9	Interviews with patients with experience of stroke approximately 4 months previously. Thematic analysis.
				Age: 64-83	
25	Alaszewski A, Alaszewski H, Potter J. 2006 [37]	Risk, uncertainty and life threatening trauma: Analysing stroke survivor’s accounts of life after stroke.	UK	n =31	Interviews with survivor or carer in individual interviews or in focus groups. Analysis based on grounded theory.
				Age: 38-89	
26	Boylstein C, Rittman M, Hinojosa R. 2007 [47]	Metaphor shifts in stroke recovery.	US	n =49	War veterans from Florida and Puerto Rico. In-depth interviews at month 1 and 6 post stroke. Grounded theory
				49 male	
27	Jones F, Mandy A, Partridge C. 2008 [40]	Reasons for recovery after stroke: A perspective based on personal experience. *Disability and Rehabilitation*.	UK	n =10	Interviewed between 6 weeks and 13 months after onset. Phenomenological approach
				6 male, 4 female	
				Mean age: 61.8	
28	Popovich JM, Fox PG, Bandagi R. [64]	Coping with stroke: Psychological and social dimensions in U.S. Patients.	US	n =60	Interviewed within the first two weeks after their stroke. Thematic analysis.
				Age: 51-89	
				Ethnicity: Black	

### Content analysis

Fifteen coded categories were identified in the preference elicitation studies (Table 4). The categories included varied across the studies, with only paralysis and dependence included in all. Only three studies made reference to continuing or worsening disability [13,14,17]. Toileting [14,35], care arrangements [24,14] and mortality [17,24] were identified in only two studies. Solomon et al. [24] included the most categories within their descriptions and this was the only study to include pain and receptive problems. The following is an example of coded text under the category “Receptive problems”:

**Table 4:** **Categories included in health state descriptions**

**Author**	**Solomon et al. [** 24 **]**	**Gage et al. [** 33]	**Hallan et al. [** 14 **]**	**Slot & Berge [** 35]	**Shin et al. [** 34 **]**	**Robinson et al. [** 13 **]**	**Samsa et al. [** 17 **]**
**Year**	**1994**	**1996**	**1999**	**2009**	**1997**	**2001**	**1998**
**Stroke severity**	**Mild/moderate severe**	**Mild/moderate/major**	**Minor/major**	**Mild/moderate/severe**	**Minor/major**	**Mild/severe**	**Major**
**Categories**							
Paralysis	x	x	x	x	x	x	x
Dependence	x	x	x	x	x	x	x
Feeling weakness- numbness, tingling	x	x	x	x	x	x	
Mobility and ambulation	x	x	x	x	x		
Expressive problems	x	x	x	x		x	x
Coordination & dexterity	x	x	x		x		
Memory/thinking	x	x	x	x			
Returning to normal activities	x	x			x	x	
Facial droop	x	x		x			
Toileting			x	x			
Care arrangements	x		x				
Mortality	x						x
Pain	x						
Receptive problems	x						
Continuing or worsening disability						x	
Number of categories	13	9	9	8	6	5	4

“You suffer a stroke that takes away your ability to understand language. You no longer understand anything being said to you” [24]

The sparest thematic content was found in Samsa et al. [17], although this only included major stroke, described as:

“a stroke that leaves an arm, a leg, and one side of your body paralyzed, and leaves you unable to take care for yourself. Anyone who has a major stroke will stay in this state until death”.

In addition and in order to help assess the relative impact of aphasia on preferences associated with major stroke, approximately 50% of the interviewees were randomly assigned to include the inability to speak in the description of the sequelae of a major stroke.

Information sources used to develop health state descriptions varied, but there was no reference to stroke patients’ perspectives; no studies included primary research with patients to ascertain them. Three studies [25,26,65] used an existing functional outcome scale – the Modified Rankin Scale (mRS) – which ranks levels of disability [50] to inform descriptions, with only one [25] citing further evidence [51] to support domains of function included. The scaling validity of the severity classifications for one study [30] were tested by neurologists specialising in stroke care. Two studies [28,29] made no reference to how the descriptions were developed. One study [27] suggested that the descriptions were adapted from a previous study [25].

Differences in content across studies could not be explained by variations in stroke severity. Of the three studies that utilised the mRS to inform the descriptions for levels of stroke severity [14,33,35]], two [33,35] provided descriptors for mild, moderate and severe/major stroke with categories common to both including: paralysis, dependence, feeling weakness, numbness or tingling, mobility and ambulation, expressive problems, memory and thinking, and facial droop. However, coordination and dexterity, and returning to normal activities, were only included in one study [33], and toileting only included in one other study [35]. Furthermore, mild/minor stroke was defined by different levels of mRS across studies (mRS = 1 [35], mRS =1-2 [33] and mRS =2-3 [14]).

Twenty nine coded categories were identified in the qualitative literature (Table 5). Counts of studies including each category showed that change in self identity and social role was the most frequently cited category (n = 26, 93%), followed by emotional difficulties (n = 25, 89%), mobility and ambulation (n = 24, 86%), and returning to normal regular activities (n = 24, 86%). The following are two examples of data from qualitative literature coded under ‘Change in self-identity, social role’:

**Table 5:** **Rank ordering of categories by counts of study and study type**

**Preference elicitation studies (n = 7)**	**Counts, (%)**	**Qualitative literature (n = 28)**	**Counts, (%)**
Paralysis	7 (100)	Change in self-identity, social role	26 (93)
Dependence i.e. feeding, dressing, washing	7 (100)	Emotional difficulties	25 (89)
Feeling weakness. numbness, tingling	6 (86)	Mobility and ambulation	24 (86)
Expressive problems	6 (86)	Returning to normal regular activities	24 (86)
Mobility and ambulation	5 (71)	Support and networks	23(82)
Coordination and dexterity	4 (57)	Coordination and dexterity	23 (82)
Memory and thinking	4 (57)	Recovery, getting better	22 (79)
Returning to normal regular activities	4 (57)	Dependence i.e. feeding, dressing, washing	20 (71)
Facial droop	3 (43)	Expressive problems	17 (61)
Toileting	2 (29)	Fatigue	16 (57)
Discharge from care and care arrangements	2 (29)	Perception by others	15 (54)
Mortality	2 (29)	Unpredictability, unreliability	14 (50)
Pain	1 (14)	Paralysis	14 (50)
Receptive problems	1 (14)	Concern for NOK	14 (50)
Continuing or worsening disability	1 (14)	Memory and thinking	13 (46)
Dizzy and faint	0 (0)	Discharge from care and care arrangements	13 (46)
Sight	0 (0)	Continuing or worsening disability	12 (43)
Fatigue	0 (0)	Perplexity	11 (39)
Mind-body split	0 (0)	Further risk	11 (39)
Loss of swallow	0 (0)	Feeling weakness. numbness, tingling	11 (39)
Concern for NOK	0 (0)	Mortality	11 (39)
Change in self-identity, social role	0 (0)	Dissociation of self and body	9 (32)
Unpredictability, unreliability	0 (0)	Dizzy and faint	6 (21)
Perplexity	0 (0)	Pain	6 (21)
Perception by others	0 (0)	Sight	6 (21)
Support and networks	0 (0)	Toileting	4 (14)
Emotional difficulties	0 (0)	Facial droop	3 (11)
Further risk	0 (0)	Loss of swallow	3 (11)
Recovery, getting better	0 (0)	Receptive problems	2 (7)

*TBW “Are you thinking about the fact that you had a stroke when you say you have changed, or are you thinking more in general?”*

*R “No, since I had the stroke. I don’t recognize myself. It is awful. You are in a way degraded. I am, even though you cannot see anything on me. Everybody says that I’m so not and so on. There’s no help in that. Nobody realizes how I am in reality.”* (Case 4) [58]

In another example loss of physical function leads a patient to struggle with his sense of who he is in conjunction with his prestroke identity, as he recounts here:

*“The one thing that’s very difficult for me as a person. . . I cannot relate, or quickly relate, back to where I was before I had the stroke. So, that comparison, I just can’t get it through my head to let that go, that I can’t do that.” (Mr. H. N.)* [55]

### Comparison of health state descriptions and patients’ experience: thematic synthesis

Mapping categories onto domains related to HRQOL for both study types resulted in four domains and associated thematic content (Table 6): biophysical (including 11 categories), mood and cognition (six categories), prognosis (four categories), and social (eight categories).

**Table 6:** **Domains and categories by counts of study and study type**

**Domains**	**Preference elicitation studies (n = 7), (%)**	**Qualitative literature (n = 28), (%)**
**Biophysical features**		
Mobility and ambulation	5 (71)	24 (86)
Coordination and dexterity	4 (57)	23 (82)
Fatigue	0 (0)	16 (57)
Paralysis	7 (100)	14 (50)
Feeling weakness- numbness, tingling	6 (86)	11 (39)
Dizzy/faint	0 (0)	6 (21)
Pain	1(14)	6 (21)
Sight	0 (0)	6 (21)
Toileting	2 (29)	4 (14)
Facial droop	3 (43)	3 (11)
Loss of swallow	0 (0)	3 (11)
**Mood and cognition**		
Emotional difficulties	0 (0)	25 (89)
Expressive problems	6 (86)	17 (61)
Memory/thinking	4 (57)	13 (46)
Perplexity	0 (0)	11 (39)
Dissociation of self and body	0 (0)	9 (32)
Receptive problems	1 (14)	2 (7)
**Prognosis**		
Getting better	0 (0)	22 (79)
Continuing or worsening disability	1 (14)	12 (43)
Further risk	0 (0)	11 (39)
Mortality	2 (29)	11 (39)
**Social features**		
Change in self-identity, social role	0 (0)	26 (93)
Returning to normal activities	4 (57)	24 (86)
Support and networks	0 (0)	23 (82)
Dependence i.e. feeding, dressing, washing	7 (100)	20 (71)
Perception by others	0 (0)	15 (54)
Unpredictability, unreliability	0 (0)	14 (50)
Concern for NOK	0 (0)	14 (50)
Discharge from care and care arrangements	2 (29)	13 (46)

Qualitative studies included a wider range of categories in every domain than health state descriptions. Health state descriptions missed categories in every domain that were relevant to patients’ experience, although all categories included in the health state descriptions were identified as important to patients in the qualitative studies . In each domain, the most often cited category differed between health state descriptions and qualitative studies and, with the exception of the biophysical domain, the most often cited categories in the qualitative studies (emotional difficulties; recovery, getting better; change in self-identity) did not appear at all in the health state descriptions.

A key feature of patients’ experience of stroke was the unanticipated, and therefore disruptive and sometimes shocking, nature of the experience. This carried through into the recovery phase, not only in the form of uncertainty about long term survival, but also in the potential for the disruption of everyday life. Categories in the biophysical domain reflect this potential disruption. Within this domain, paralysis was cited most often in the descriptions, in contrast to mobility and ambulation in the qualitative studies. Furthermore, dizziness and fainting, effects on sight, loss of swallow and fatigue, that were prominent in the qualitative literature, were absent from health state descriptions. Most notably, fatigue was cited in over half of the qualitative studies and the following are two examples of text coded under this category:

*“Such a small and simple thing that you used to do in no time at all without even thinking, you, well, you now have to put all your energy into it… and also when you have to carry something in, you sort of feel how useless it is* (I, male 59 years, married)” [53]

*“This feeling of fatigue, it comes as quick as a bolt of lightning. I don’t feel any signals, and all of a sudden I’m totally exhausted. I should have a timer that tickled me every hour, so I know that I should stop and take a rest”* [48]

Within the mood and cognition domain, emotional difficulties, dissociation of self and body, and perplexity were absent from all health state descriptions, despite their prominence in the patient perspective. In particular, emotion was the most cited category from qualitative studies, and one of the most cited categories overall in the qualitative literature, whereas expressive problems were most often cited in the health state descriptions. Examples of text coded under ‘emotion’:

*“I thought that it could not be true! I felt desperate because of what had happened. I thought that it could not be true, so I tried to walk, but I couldn’t. . . . I became very depressed and cried a lot.”* [42]

Overall, an alphabet of feelings was mentioned: angry, ashamed, bewildered, burdensome, depressed, frustrated, helpless, inadequate, imperfect, shocked, suicidal, surprised, tearful, tetchy, traumatized, vulnerable, worried:

*“ this is why I’m so frustrated - everything I do, I’m so slow to what I used to be.”* [60]

Within the prognosis domain, concerns about recovery and further risk were prominent in the patient perspective but missing from the health state descriptions. In addition, a wider range of categories relevant to patient experience in the social domain were absent from health state descriptions than any other domain. These included unpredictability and unreliability, concern for next of kin, perception by others, support and networks, and changes in self-identity and social role; the last two were cited most often in qualitative studies, whereas dependence was dominant in the health state descriptions.

## Discussion

To our knowledge, this is the first study to explore the extent to which stroke health state descriptions used in preference elicitation studies reflect patients’ experience, by reviewing and comparing published health state descriptions used in elicitation studies with a qualitative synthesis of stroke patients’ perspectives on what is important to them. Variation exists in the content of health state descriptions for all levels of stroke severity. Of greater concern is the major disjuncture between how stroke is experienced by patients and the representation of stroke in the health state descriptions, which appears to reflect an absence of engagement of stroke patient perspectives in their development. This raises significant concerns about the validity of the descriptions and hence the values derived from them, and about the methods used to develop health state descriptions. This has potentially significant consequences for the use of the values elicited using such descriptions in health policy and clinical/patient decisions.

There are some study limitations. Despite covering a range of different strokes and stroke severities [24,48,55,60,61,63], the available accounts tend to exclude the perspectives of those with more severe strokes, particularly involving speech impairments [48]. Nonetheless, our review included a wide range of studies and patient groups. In order to indicate the spread of categories across the data sources, we counted the number of data sources (published qualitative studies and health state descriptions) in which these categories appear. This is an indirect measure of importance to patients, but nonetheless captures the presence of key categories across a range of studies exploring patient experience. An inherent limitation of content analysis is that counts of content cannot in itself produce a deep understanding of the data, but to ameliorate this we also conducted and report a thematic synthesis.

Previous research regarding the impact of the measurement process on utility values exists. Specifically for stroke, variations in estimates resulting from direct preference elicitation have previously been explained by a number of factors. First, the choice of study population; for example, healthy participants assign lower utilities than patients who have experienced stroke [11,17]. Second, the method of elicitation, with standard gamble derived scores being generally higher than those derived from time trade off studies, which in turn are higher than those derived from visual analogue scales [16,18]. Third, the bounds of the scale, typically 0–1, may be defined differently: the upper bound defined as perfect health yields lower utility scores than if defined as the absence of the condition of interest, which is no guarantee that health is perfect [19].

Variations in health state description content may also explain differences. The level of detail in health state descriptions can affect utility scores; longer, more comprehensive, descriptions (although appearing to have more face validity), may overload respondents’ cognitive capacity, so that they latch onto a few key phrases and ignore the rest [20]. Naming or labelling a condition may have an impact [10,12,21-23] with a recent study recommending avoiding condition labels in health state descriptions to ensure that values are not affected by prior knowledge or preconceptions of the condition that may distort the health state being valued [10]. Furthermore, the wording may also cause variation in values if not presented in a balanced manner (framing bias), with both positive and negative effects described; explicit inclusion of negative aspects of stroke elicit lower values [24,25].

A key element in developing valid health state descriptions is whether the description accurately reflects patient experience. However, there is little empirical work on the content validity of health state descriptions, nor on the methods of their development, despite long standing arguments for this [22,66]; this may explain significant variations or biases in utility scores [67]. This failure to take account of patient experience is the most probable explanation for the observed variation in stroke health state descriptions, and most importantly for the disjuncture between them and what is important to patients. At a population level, variations or biases in utility scores may have serious implications for resource allocation decisions within health care systems. For example, NICE recommends the use of Quality Adjusted Life Years (QALYs) as a measure of health benefit for their ‘reference case’, to enable a standardized approach for comparing economic evaluations across different healthcare areas [1]. Indirect preference elicitation using the EQ-5D is the method and measure of HRQOL in adults that is preferred by NICE decisions taken at a national level. Despite this, a review of the selection and use of health-related utility values for economic models included in NICE Technology Appraisals [68] found that only 56% of submissions to NICE and assessment reports included utility values that met the relevant reference case. This highlights variation in the methods used to select and incorporate utility values in economic models for NICE Technology Appraisals. Furthermore, methods for guideline production in other countries are in general less prescriptive regarding methods of preference elicitation [69], thus being more likely to incorporate direct methods of preference elicitation. The use of direct preference elicitation methods where health state descriptions may lack content validity could have an impact on the estimated cost effectiveness of health interventions and associated resource allocation decisions.

Health state descriptions were commonly derived from the Modified Rankin Scale (mRS), a clinician-derived measure of global disability [70], but the content of these descriptions differed across studies. Descriptions in other studies were either derived directly from clinicians or their derivation was not described. Most critically, none of the preference elicitation studies utilised stroke patients’ perspectives to inform the content of the descriptions, in stark contrast to the standard methods of developing HRQOL measures [71], where the extent to which patient experience has generated the content and domains is a critical indicator of validity [18,19].

These findings are disturbing - nearly two decades ago it was recommended that health state description development should draw upon a range of perspectives, by collating information on health states by using the evidence base, and/or interviewing medical professionals and patients, in order to seek a consensus on the most important aspects of quality of life and their relative importance [72]. Similarly, the importance of incorporating patients’ perspectives to establish domain and content validity of the impact of stroke on QOL using qualitative research, either by asking patients directly or by using the evidence base has also been recognised [9]. Despite these recommendations, none of the preference elicitation studies for stroke used these methods.

Given this absence of the patient perspective, the disjuncture between how stroke is experienced and the representation of stroke in the health state descriptions is not surprising. As detailed in the results above, for example, the qualitative studies demonstrate that unresponsive or unpredictably responsive limbs, fatigue, cognitive difficulties and emotional ability led those recovering from stroke to curtail their activities, resulting for many in lives that little resembled their pre-stroke existence. This contrasts with the lack of emphasis on these features in health state descriptions. Furthermore, the effects of stroke on everyday life had significant implications for social role, identity and relationships. No longer able or confident in their ability to engage in everyday activities, the relationships associated with these activities shifted. Stroke survivors often disengaged from, or restricted, their social networks, leading to social isolation. Social relationships were disrupted, via dependence on others, resulting from the physical and emotional effects of stroke. These social characteristics were noticeably absent from the health state descriptions.

Whilst direct methods of preference elicitation have an important role to play, the content validity of health state descriptions for stroke, as with HRQOL instruments, can only be established if patients’ perspectives on the impact of the health state are incorporated into their development. The fact that patients’ experiences of stroke incorporate a much wider set of categories than those incorporated in the health state descriptions, emphasises the importance of this.

## Conclusions

Key features of the methodological process for directly eliciting utility values for stroke can explain variations in estimates. One such feature is the design and content of health state descriptions. Our findings not only show that there is no systematic method for constructing the content/scope of health state descriptions for stroke, but also critically that the perspective of patients is not incorporated. We have demonstrated that this produces descriptions with major deficits in reflecting the lived experience of stroke, and raises serious questions about the values derived from such descriptions, which might lead to erroneous conclusions in decisions made based on these values.

We recommend that health state descriptions used for direct preference elicitation, as with HRQOL instruments, should be developed with reference to patient perspectives derived from published qualitative research and/or directly from patients themselves. Further research into the differing impact of descriptions that do or do not incorporate what is important to patients would help to characterise the impact of these deficits in terms of utility scores and associated Quality Adjusted Life Years (QALYs) and resource allocation decisions.

### Ethics statement

Ethical approval was not needed for this study as it is based upon two literature reviews and involves no human contact.
